# Radiation exposure in endovascular stroke treatment of acute basilar artery occlusions—a matched-pair analysis

**DOI:** 10.1007/s00234-020-02490-0

**Published:** 2020-07-10

**Authors:** Charlotte S. Weyland, Arne Potreck, Ulf Neuberger, Markus A. Möhlenbruch, Simon Nagel, Peter A. Ringleb, Martin Bendszus, Johannes A. R. Pfaff

**Affiliations:** 1 Department of Neuroradiology, Heidelberg University Hospital, Im Neuenheimer Feld 400, 69120 Heidelberg, Germany; 2 Department of Neurology, Heidelberg University Hospital, Im Neuenheimer Feld 400, 69120 Heidelberg, Germany

**Keywords:** Radiation exposure, Fluoroscopy, Endovascular stroke treatment, Basilar artery

## Abstract

**Abstract:**

**Purpose:**

To determine the radiation exposure in endovascular stroke treatment (EST) of acute basilar artery occlusions (BAO) and compare it with radiation exposure of EST for embolic middle cerebral artery occlusions (MCAO).

**Methods:**

In this retrospective analysis of an institutional review board−approved prospective stroke database of a comprehensive stroke center, we focused on radiation exposure (as per dose area product in Gy × cm^2^, median (IQR)), procedure time, and fluoroscopy time (in minutes, median [IQR]) in patients receiving EST for BAO. Patients who received EST for BAO were matched case by case with patients who received EST for MCAO according to number of thrombectomy attempts, target vessel reperfusion result, and thrombectomy technique.

**Results:**

Overall 180 patients (*n* = 90 in each group) were included in this analysis. General anesthesia was conducted more often during EST of BAO (BAO: 75 (83.3%); MCAO: 18 (31.1%), *p* < 0.001). Procedure time (BAO: 31 (20–43); MCAO: 27 (18–38); *p* value 0.226) and fluoroscopy time (BAO: 29 (20–59); MCAO: 29 (17–49), *p* value 0.317) were comparable. Radiation exposure was significantly higher in patients receiving EST for BAO (BAO: 123.4 (78.7–204.2); MCAO: 94.3 (65.5–163.7), *p* value 0.046), which represents an increase by 23.7%.

**Conclusion:**

Endovascular stroke treatment of basilar artery occlusions is associated with a higher radiation exposure compared with treatment of middle cerebral artery occlusions.

## Introduction

In the wake of the recent establishment of endovascular stroke treatment (EST) as a standard of care for patients with ischemic strokes and large vessel occlusions, the number of interventions has been increasing in the last years and will continue to rise [1, 2]. With an expanding network of neurointerventional units performing EST, the demand for procedural quality standards has increased as well [3].

While in diagnostic radiology, radiation exposure (RE) is a key concern of physicians and technicians, especially regarding the innovation of CT imaging, RE during EST is not well investigated so far. Following the ALARA (“as low as reasonably achievable”) principle the interventionalists should be aware of RE during the procedure and the necessity for protection of involved staff and themselves as well as the minimization of stochastic risks and possible deterministic risk for the patient [4, 5]. Of course, RE will not influence the decision-making process before or during EST, but a systematically high RE in individual interventional units or connected to an interventionalist might represent a characteristic of treatment quality.

Systematically high RE could be detected, if adequate reference levels for radiation exposure during EST were available. Comparably, diagnostic reference levels in diagnostic radiology have already proven to effectively control and reduce RE during CT-imaging [4, 6]. Recently, a more thorough investigation of RE during EST has begun [7]. Individual cases of EST can be associated with unusually high radiation exposure, e.g., due to complications or difficult vascular access [8]. Also, unexperienced interventionalists cause a higher radiation exposure during EST [9], a fact that implies the need for (simulator-)training and awareness among interventionalists. Software adjustments to reduce RE are an effective measure, that is already implied in many stroke centers [10]. Biplane angiographic systems show a reduced fluoroscopy time, contrast load, and RE compared with monoplane systems, while the choice of system does not influence the technical or clinical outcome of EST [11].

When investigating RE of EST, a question at hand is whether RE for EST of the anterior and posterior circulation is significantly different. The procedural setup with a different anatomic vessel pathway and aspects of imaging (e.g., field of view) are not identical. We hypothesized that RE during EST significantly differs between the anterior and the posterior circulation. Correspondingly, the objective of this study was to compare RE of EST in the anterior and posterior circulation.

## Methods

This article reports data from a retrospective analysis of a prospectively maintained, institutional review board−approved stroke database. All data reported originate from a university based comprehensive stroke center. The analysis focuses on RE, procedure time, and fluoroscopy time in EST of the posterior circulation involving acute, basilar artery occlusions (BAO) and EST of the anterior circulation involving acute occlusions of the main branch of the middle cerebral artery (MCAO).

### Patient treatment

Decision for EST was made on a consensus by the treating neurologist and neurointerventionalist based on current national and international guidelines. Initial imaging (CT/MRI) showed the BA-/MCA-occlusion, excluded hemorrhage, and proved the presence of salvageable tissue.

EST was performed through a transfemoral arterial access in all cases. As a standardized approach a guide catheter was placed within the proximal parent artery, i.e., either the subclavian artery for BAO (7F/90 cm Flexor Shuttle, Cook Medical, Bloomington, IN, USA) or the common carotid artery for MCAO (8F VistaBriteTip, Cordis, Santa Clara, CA, USA). A distal access catheter was then introduced to the vertebral artery or the internal carotid artery (Sofia 5F or Sofia Plus 6F, MicroVention, Aliso Viejo, CA, USA). The first line approach (performing direct thromboaspiration or stent-retriever-thrombectomy in combination with continuous distal aspiration using a distal access catheter), as well as the choice of material used for the EST (e.g., catheters, (micro-) guidewires, and stent-retriever), was at the discretion of the treating neurointerventionalist.

All patients were treated using a biplane angiographic system (Artis Zee biplane or Artis Q, Siemens Healthineers, Germany). The angiography systems underwent technical surveillance with repetitive constancy tests according to German Institute for Standardization (Deutsches Institut für Normung e.V. = DIN) standards [12, 13] for quality assurance and to comply with legal regulations concerning RE. Operation of the angiographic system and optimization of exposed area during fluoroscopy was done by the interventionalist. Contrast runs to display the anterior circulation in cases with BAO or the posterior circulation and contralateral ICA territory in MCAO before the first thrombectomy attempt to visualize collateralization were not performed. A thrombectomy attempt was defined as a planned and conducted maneuver with the intention to recanalize an occluded intracranial vessel.

### Inclusion and exclusion criteria

All patients treated with EST for a thromboembolic BAO between January 2013 to December 2019 were included in this study. Patients who received EST for BAO were matched case by case with patients who received EST for a thromboembolic MCAO according to reperfusion result, number of thrombectomy attempts, and thrombectomy technique for each thrombectomy attempt (stent-retriever thrombectomy under continuous aspiration vs. direct thromboaspiration) [8, 14]. Patients receiving a (stent-assisted) balloon-angioplasty of the parent or occluded vessel were excluded from the analysis due to expected associated higher procedure time, fluoroscopy time, and RE. We excluded all procedures performed as one of the first 25 EST-procedures of the involved interventionalists because of the known interventionalist’s learning curves [9, 15]. Patients who received i.a. thrombolysis only, cases of target vessel recanalization following IVT, and cases without thrombectomy attempt (e.g., due to futile vascular access) were excluded as well. The clinical status of the patient on admission and the choice of sedation mode during EST were not established as matching criteria in this analysis as they are not influencing the RE [16].

### Primary outcome parameters

Primary outcome parameters were procedure time (defined and calculated from time of groin puncture to time of last angiographic image), fluoroscopy time (defined as total amount of fluoroscopy time from both—lateral and posterior-anterior—detectors of the biplane angiographic system)—both time metrics in minutes—and RE as per dose area product (DAP) in Gy × cm^2^.

### Data acquisition

Data in our database were prospectively acquired using medical exams, procedural reports of the interventionalist, automatically generated procedure protocols of the angiographic systems, and obtained angiographic images. For this analysis, data such as patient’s age, sex, date, and time of stroke onset were retrieved from the database. Additionally, data concerning procedural aspects such as mode of anesthesia during intervention (conscious sedation or general anesthesia), use of stent-retrievers for mechanical thrombectomy or direct thromboaspiration, number of thrombectomy attempts, and post-interventional reperfusion result as per modified thrombolysis in cerebral infarction (mTICI) were retrieved. To compare reperfusion results in the posterior circulation with mTICI in MCAO, we scored partial reperfusion (for example, a remaining occlusion of a superior cerebellar artery or any occlusion in a posterior cerebral artery) as mTICI 2b and complete reperfusion as mTICI 3. Procedure time, fluoroscopy time, and DAP were taken from exam protocols.

### Statistical analysis

Data are shown as median with interquartile range (IQR) or means with standard deviation (SD), as appropriate Mann–Whitney *U* test, two-sided *t* tests, and two-sided Chi-square tests were used to compare groups after testing for normal distribution with the Shapiro–Wilk test. A two-sided level of significance with a *p* value of less than or equal to 0.05 was considered to indicate a significant difference. All statistical analyses were performed by using SPSS (IBM, Armonk, NY).

## Results

Between January 2013 and December 2019 EST for BAO was performed in *n* = 166 patients. Of these, *n* = 90/166 (54.2%) patients fulfilled inclusion and exclusion criteria and entered the analysis. Only a few deviations from precise matching were made: (i) in two patients with BAO in who no substantial reperfusion was achieved were matched with patients in who mTICI 2b was reached during EST of MCAO, (ii) one patients with BAO in who complete reperfusion was achieved was matched with a patient in who mTICI 2c was achieved during EST of MCAO, and (iii) one patient requiring two attempts (1 × stent-retriever-thrombectomy and 1 × aspiration thrombectomy) for treatment of BOA was matched with a patient who received two aspiration thrombectomy attempts for MCAO. Other than that, all patients were exactly matched case by case with patients treated for MCAO during the same period—see Table 1.

**Table 1:** Baseline characteristics and procedural aspects of patients who received endovascular stroke treatment for thromboembolic basilar artery occlusion and matched patients who received endovascular stroke treatment for middle cerebral artery occlusion in the observation period from January 2013 to December 2019

		BA occlusion (*n* = 90)	MCA occlusion (*n* = 90)	*p* value
Age (year), median (IQR)		76 (68–82)	78 (67–84)	0.219‡
Male (%)		40 (44)	26 (29)	0.065†
Unknown time of symptom onset (%)		29 (32.2)	30 (33.3)	0.874†
Time from stroke onset^a^ to final reperfusion result, in minutes, median (IQR)		375 (260–526.5)	332 (235–576)	0.739‡
Procedural aspects				
Treatment in general anesthesia, (%)		75 (83.3)	28 (31.1)	< 0.001†
Procedure Time (groin puncture to target vessel reperfusion), in minutes, median (IQR)		31 (20–43)	27 (18–38)	0.226‡
Dose area product, in Gy × cm^2^, median (IQR)		123.4 (78.7–204.2)	94.3 (65.5–163.7)	0.046‡
Fluoroscopy time, in minutes, median (IQR)		29 (20–59)	29 (17–49)	0.317‡
Thrombectomy attempts^b^				
Thrombectomy attempts in total, median (IQR)		2 (1–3)	2 (1–3)	0.928†
Patients with aspiration thrombectomy only, *n* (%)		18 (20.0)	19 (21.1)	
Thrombectomy attempts with aspiration, median (IQR)		0 (0–1)	0 (0–1)	
Patients with stent-retriever thrombectomy only, *n*		67 (74.4)	67 (74.4)	
Thrombectomy attempts with stent-retriever thrombectomy, median (IQR)		1 (1–3)	1 (1–3)	
Final angiographic reperfusion result^b^				
BA occlusion		MCA occlusion		0.853†
No substantial reperfusion, *n* (%)	11 (12.2)	mTICI 0–2a, *n* (%)	9 (10)	
Partial reperfusion, *n* (%)	31 (33.4)	mTICI 2b, *n* (%)	29 (32.2)	
		mTICI 2c, *n* (%)	5 (5.6)	
Complete reperfusion, *n* (%)	48 (53.3)	mTICI 3, *n* (%)	47 (52.2)	

^a^Or time of last seen well

^b^Matching criterion

†Chi-square test, two-sided

‡Mann–Whitney *U* test

While the age distribution was comparable in the two sub-groups (median (IQR): BAO: 76 (68–82); MCA0: 78 (67–84), *p* value: 0.219), patients treated for BAO (*n* = 40 (44%)) tended to be more often male than patients treated for MCAO (*n* = 26 (29%), *p* = 0.065). The time from stroke onset to final reperfusion result was comparable. Treatment in general anesthesia as opposed to conscious sedation was significantly more often performed for patients with BAO (BAO: *n* = 75 (83.3%); MCAO: *n* = 28 (31.1%), *p* < 0.001) (for details also see Table 1).

### Primary outcome parameters

Procedure time was comparable between patient groups (BAO: 31 (20–43); MCAO: 27 (18–38); *p* value 0.226). Fluoroscopy time was comparable between the two groups as well (BAO: 29 (20–59); MCAO: 29 (17–49), *p* value 0.317).

RE was higher during EST of acute basilar artery occlusion than during EST of occlusions of the main branch of the middle cerebral artery (BAO: 123.4 (78.7–204.2); MCAO: 94.3 (65.5–163.7), *p* value 0.046). This reflects a relative increase of 23.7% of RE in patients who received EST for BAO.

## Discussion

As a main result, this study shows a higher RE during EST of BAO compared with occlusions of the main branch of the middle cerebral artery. This is especially noteworthy as procedure time and fluoroscopy time do not differ among patient groups. The matched pair analysis by number of thrombectomy attempts, thrombectomy technique, and angiographic reperfusion result eliminate firstly the confounder, that BAO might be treated more often by stent-retriever thrombectomy, which is associated with a higher RE [14]. Secondly the difference in RE can thus not be explained by a higher number of thrombectomy attempts or better recanalization rate in one of the study groups. Interventions with a comparably inexperienced neuro-interventionalist in charge were also excluded from this analysis for the known effect on experience on lowering the RE [9].

Patients treated for BAO were as expected more often treated in general anesthesia than patients with MCAO, which is primarily due to the higher possibility of compromised level of consciousness associated with BAO. It was, however, shown previously that the mode of anesthesia is negligible when comparing RE during EST. [16] The selected mode of sedation was therefore not considered as matching criterion, and it is rather doubtful that it should have confounded RE in the current analysis.

In the few studies on RE in EST at hand, procedures of the posterior circulation were either excluded [17], not separately analyzed or not sufficiently represented in the patient cohort [18]. Farah et al. offer an analysis of RE in EST on a patient cohort of *n* = 319 patients. This cohort only includes 5.1% of procedures in the posterior circulation (*n* = 16) [7]. The current article contradicts Farah et al., who stated that no relevant difference of RE during EST for the anterior and posterior circulation was seen in their patient cohort. Farah et al. also acknowledge that the very low case number for EST in the posterior circulation could be a possible confounder. RE of EST for acute MCAOs and for BAOs as reported in the current manuscript are both within the range of the overall RE reported previously [7, 9, 16]. However, we could demonstrate a substantial difference and a 23.7% increase of RE for EST in BAOs compared with EST for MCAO. This means that a lower range for reference levels of the anterior circulation opposed to a higher reference level for EST in posterior circulation large vessel occlusions seems more accurate.

Since this study eliminated confounders that influence the RE during EST, the observed higher RE for EST of BAO can be explained by differences in anatomical access or angiography settings. The subclavian arteries and origin of the vertebral arteries lying between shoulder and clavicle, i.e., surrounded by more soft and osseous tissue, compared with the more exposed position of the cervical carotid bifurcation, are an anatomical difference at hand. If and how this really influences the RE is still uncertain. Secondly, the position and angulation of the c-arms of the biplane angiographic system and the field of views are different for BAO and MCAO. Due to the focus on the posterior fossa, the scull base is more prominently exposed to x-rays in BAO, a fact that can contribute to higher RE necessary for image quality comparable to the MCAO.

The suggestion of reference levels by Guenego et al., who investigated RE in EST with an additional dose reduction system in a retrospective multicenter-study with *n* = 1096 patients, did not differentiate between anterior and posterior circulation procedures as well [10]. The previously reported RE for EST (148 Gy × cm^2^ as a reference level for any EST-procedure and 114 Gy × cm^2^ with an applied dose reduction software for mixed patient cohort including BAO and MCAO [10]) are very similar to values reported in the current manuscript. Thus, the pursued reference levels for RE in EST of the anterior circulation can be held lower than for the posterior circulation. However, data on RE in BAO are still limited; thus, a clear specification of thresholds for RE in BAO appears premature and further data collection involving different comprehensive stroke centers, interventional techniques, angiographic systems of different vendors, and monoplane and biplane angiographic systems appears indispensable.

### Limitations

This study’s limitations are mainly due to the retrospective, single-center character. RE in EST might differ between different stroke centers as angiography units and thrombectomy technique differ as well. Patient’s anatomic variants and bodies (including body mass index; no-necked, short-necked, and long-necked patients) are not considered in this study [19, 20]. If this plays a role in the differing EST of the anterior and posterior circulation remains uncertain until further investigation.

## Conclusion

In this matched-pair analysis a significantly higher RE was demonstrated in endovascular stroke treatment of acute basilar artery occlusions compared with endovascular stroke treatment for occlusions of the middle cerebral artery. When defining dose reference levels for endovascular stroke treatment, establishing individual reference levels for the anterior and posterior circulation should be discussed.

## Data Availability

Individual participant data that underlie the results reported in this article (after deidentification) will be available to researchers who provide a methodologically sound proposal. Proposals should be directed to the corresponding author beginning 24 months and ending 48 months following article publication.

## Abbreviations

ALARA: As low as reasonably achievable
BAO: Basilar artery occlusion
DAP: Dose area product
EST: Endovascular stroke treatment
MCAO: Middle cerebral artery occlusion
mRS: Modified Rankin scale
NIHSS: National Institutes of Health Stroke Scale
RE: Radiation exposure
